# Endoscopic resection of adult sphenoid sinus myofibroma: a rare case report with narrative review

**DOI:** 10.3389/fonc.2026.1807570

**Published:** 2026-04-16

**Authors:** Chong Zhang, Zhipeng Wu, Bing Chen, Yuyang Zhao, Yujing Zhao, Zhe Wang

**Affiliations:** 1 School of Clinical Medicine, Shandong Second Medical University, Weifang, Shandong, China; 2 Department of Neurosurgery, Weifang People’s Hospital, Shandong Second Medical University, Weifang, Shandong, China

**Keywords:** adult, case report, endoscopic surgery, myofibroma, sphenoid sinus

## Abstract

This study documents an exceptionally rare instance of primary myofibroma originating in the sphenoid sinus of a 60-year-old female patient, who presented with acute progressive diplopia, ptosis, and headache. Preoperative imaging identified a hypervascular lesion within the sphenoid sinus, accompanied by significant bone destruction. The patient underwent successful gross total resection via an endoscopic endonasal transsphenoidal approach, which enabled complete tumor excision, preservation of vital neurovascular structures, and effective skull base reconstruction. Postoperatively, the patient’s neurological deficits improved significantly without complications. Histopathological and immunohistochemical evaluations, which were positive for smooth muscle actin (SMA) and Vimentin, confirmed the diagnosis. The tumor’s high-risk features, such as its deep location, bone infiltration, and a 15% Ki-67 proliferation index, prompted a postoperative multidisciplinary team to consider adjuvant radiotherapy due to the increased risk of local recurrence. This case demonstrates that endoscopic resection is a safe and effective primary treatment for this rare condition and underscores the importance of individualized risk assessments and a multidisciplinary approach in managing high-risk sphenoid sinus myofibroma in adults.

## Introduction

Myofibroma is a benign neoplasm originating from myofibroblasts (1). Its incidence demonstrates a notable age-related disparity, predominantly affecting infants, children, and adolescents, potentially due to germline or somatic mutations in the PDGFRB gene (2). Myofibroma occurrence in adults is rare, and primary myofibroma arising in the sphenoid sinus cavity is exceedingly uncommon, with no precise epidemiological data available; only sporadic case reports of infantile myofibroma in regions adjacent to the sphenoid sinus have been documented (3). This case may represent one of the first documented instances of primary adult sphenoid sinus myofibroma. Given the proximity of the sphenoid sinus to critical anatomical structures such as the optic nerves, internal carotid arteries, cavernous sinuses, and pituitary gland, tumors in this region often present with non-specific symptoms, including headache, visual impairment, and diplopia, and can readily lead to serious complications (4). Surgical gross total resection is the preferred treatment modality, with recurrence rates significantly associated with the extent of resection, and a lower recurrence rate observed following complete removal (5, 6). This article reports a case of primary adult sphenoid sinus myofibroma treated with gross total resection via endoscopic neurosurgery. It aims to provide a detailed description of the clinical, imaging, and pathological characteristics of this rare entity, elucidate the technical nuances and safety profile of the endoscopic surgical approach, and discuss the postoperative multidisciplinary therapeutic decision-making process based on high-risk pathological features, such as a high Ki-67 proliferation index, hoping to offer a reference for the standardized management of this disease.

## Case report

A 60-year-old female patient was admitted to the hospital due to “diplopia for 8 days, accompanied by headache and dizziness for 5 days.” Eight days prior to admission, the patient developed diplopia without an obvious precipitating cause, accompanied by right eyelid ptosis. Five days prior, she experienced intermittent distending pain in the frontal and parietal regions along with dizziness, and had one episode of vomiting. Neurological examination revealed right upper eyelid ptosis. The left eye was in an abducted position with impaired adduction. Visual field testing showed deficits in the temporal and superior quadrants of the right eye and in the superior quadrant of the left eye.

### Imaging studies

**Figure 1:**
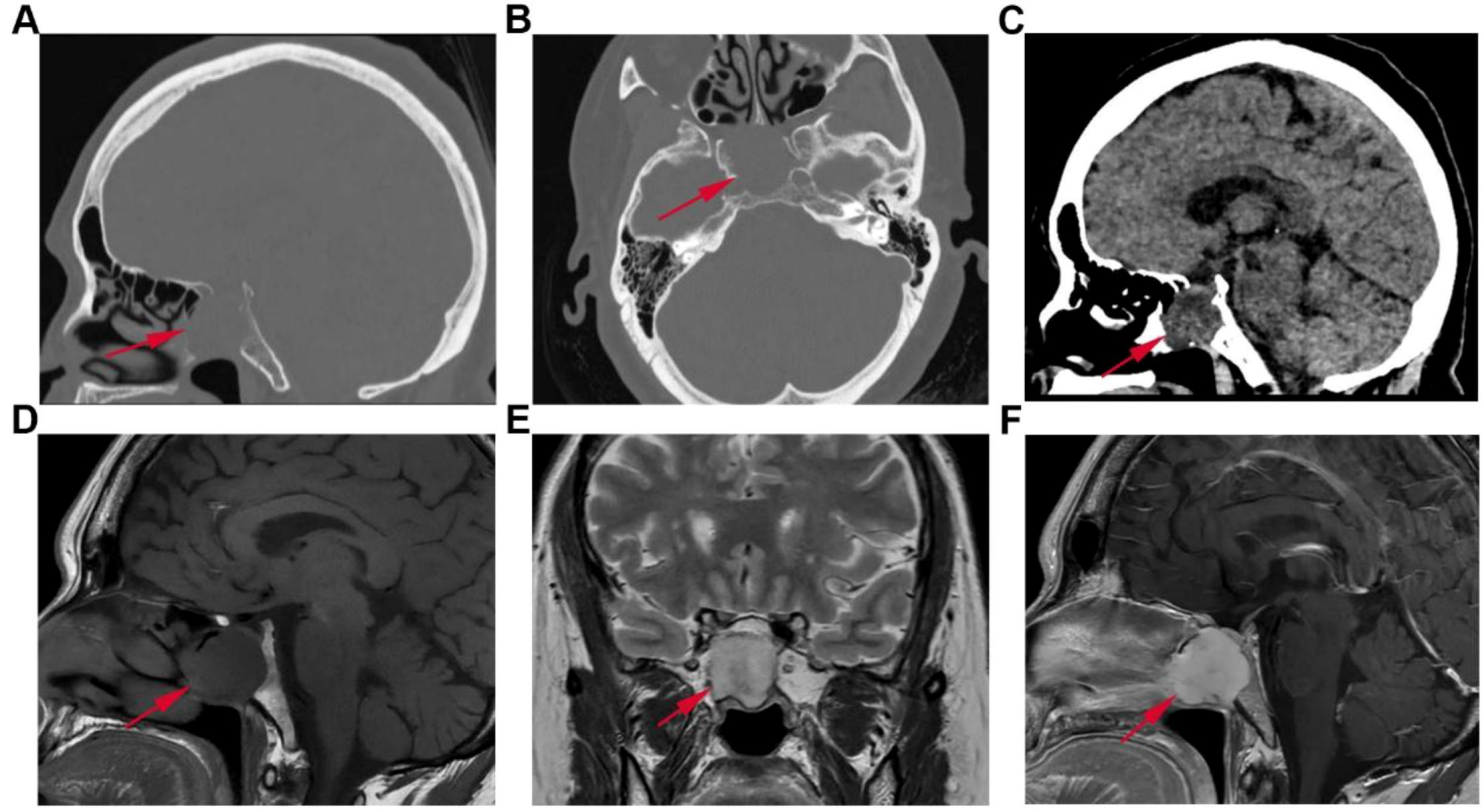
Preoperative imaging studies of the patient. **(A–C)** Cranial CT images: **(A)** Sagittal bone view. **(B)** Axial bone view. **(C)** Sagittal brain view. **(D–F)** Cranial MR images: **(D)** Sagittal T1-weighted image. **(E)** Coronal T2-weighted image. **(F)** Sagittal T1-weighted contrast-enhanced scan.

After admission, relevant auxiliary examinations were completed. Cranial computed tomography (CT) scans revealed an enlarged sella turcica with partial absence of the sellar floor bone (**Figures 1A, B**), and a round-like soft tissue density shadow was observed within the sphenoid sinus (**Figure 1C**). Magnetic resonance imaging (MRI) plain scan and dynamic contrast-enhanced sequences showed a round-like lesion in the sphenoid sinus region exhibiting long T1 and long T2 signals, with adjacent bone resorption and destruction and discontinuity of the sellar floor bone (**Figures 1D, E**). Contrast-enhanced scans demonstrated marked enhancement of the lesion and compression of the pituitary gland (**Figure 1F**).

### Treatment course

**Figure 2:**
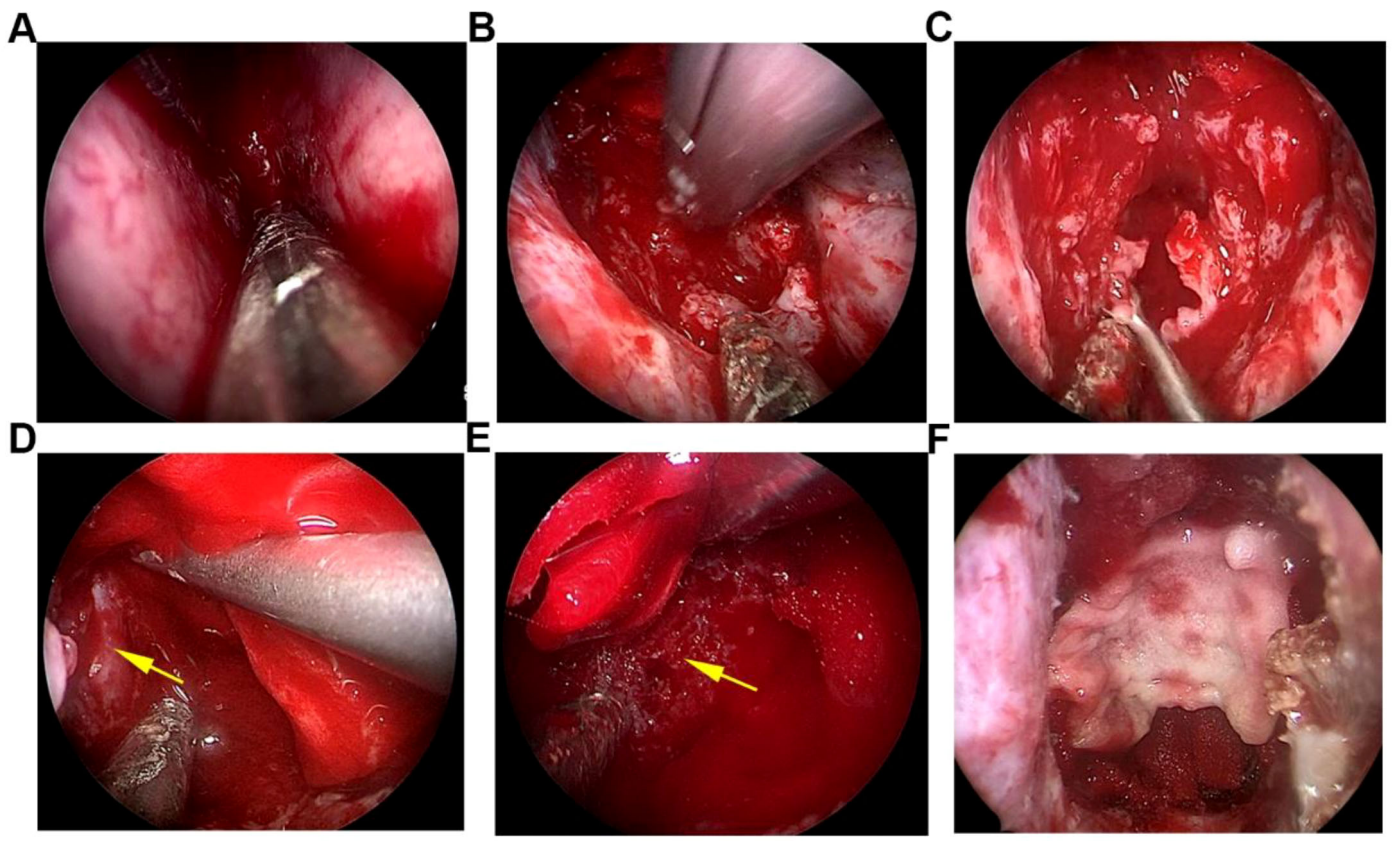
Intraoperative endoscopic views during the endonasal endoscopic resection of the sphenoid sinus myofibroma. **(A)** Endoscopic endonasal approach. **(B)** Hypervascular tumor within the sphenoid sinus. **(C)** Intratumoral piecemeal resection and decompression. **(D)** Exposed right internal carotid artery. **(E)** Tumor-eroded sellar floor and clival bone. **(F)** Artificial dura mater reinforcement and reconstruction of the surgical area.

Following intra-departmental discussions and a multidisciplinary team (MDT) consultation, a differential diagnosis of either a malignant tumor originating from the sphenoid sinus or an invasive pituitary adenoma was established. The tumor’s substantial mass effect, coupled with progressive neurological dysfunction, necessitated surgical intervention. The patient underwent an “endoscopic resection of the skull base (sphenoid sinus) lesion with dural patch repair” under general anesthesia with endotracheal intubation. Intraoperatively, utilizing a nasal approach, significant erosion of the anterior wall of the sphenoid sinus by the tumor was observed. The residual anterior wall was drilled away to fully expose the sinus cavity. The tumor within the sinus cavity was characterized by a grayish-red coloration, firm texture, and marked hypervascularity, with dense adhesion to the sphenoid sinus mucosa (**Figures 2A, B**). A piecemeal resection was conducted to achieve internal decompression of the tumor (**Figure 2C**). During this process, significant bleeding was encountered and meticulously controlled using an ablation electrode. Dissection along the tumor’s border revealed erosion of the sellar floor and clivus bones, which exhibited a moth-eaten appearance (**Figure 2E**). Additionally, a partial bone defect of the right internal carotid artery canal wall was identified, with the artery exposed yet pulsating adequately, necessitating careful protection (**Figure 2D**). The tumor exhibited dense adherence to the outer layer of the sellar dura. Through precise dissection and resection, a gross total resection of the tumor was accomplished under endoscopic visualization. Following meticulous hemostasis within the surgical cavity, the operative field underwent repeated irrigation with warm saline. Due to the partial defect in the outer layer of the sellar dura, reinforcement was achieved using an artificial dura mater patch (**Figure 2F**). The reconstruction of the cranial base was further enhanced with an absorbable dural sealant medical adhesive. Lastly, an iodoform gauze pack was placed in each nasal cavity.

### Postoperative course

**Figure 3:**
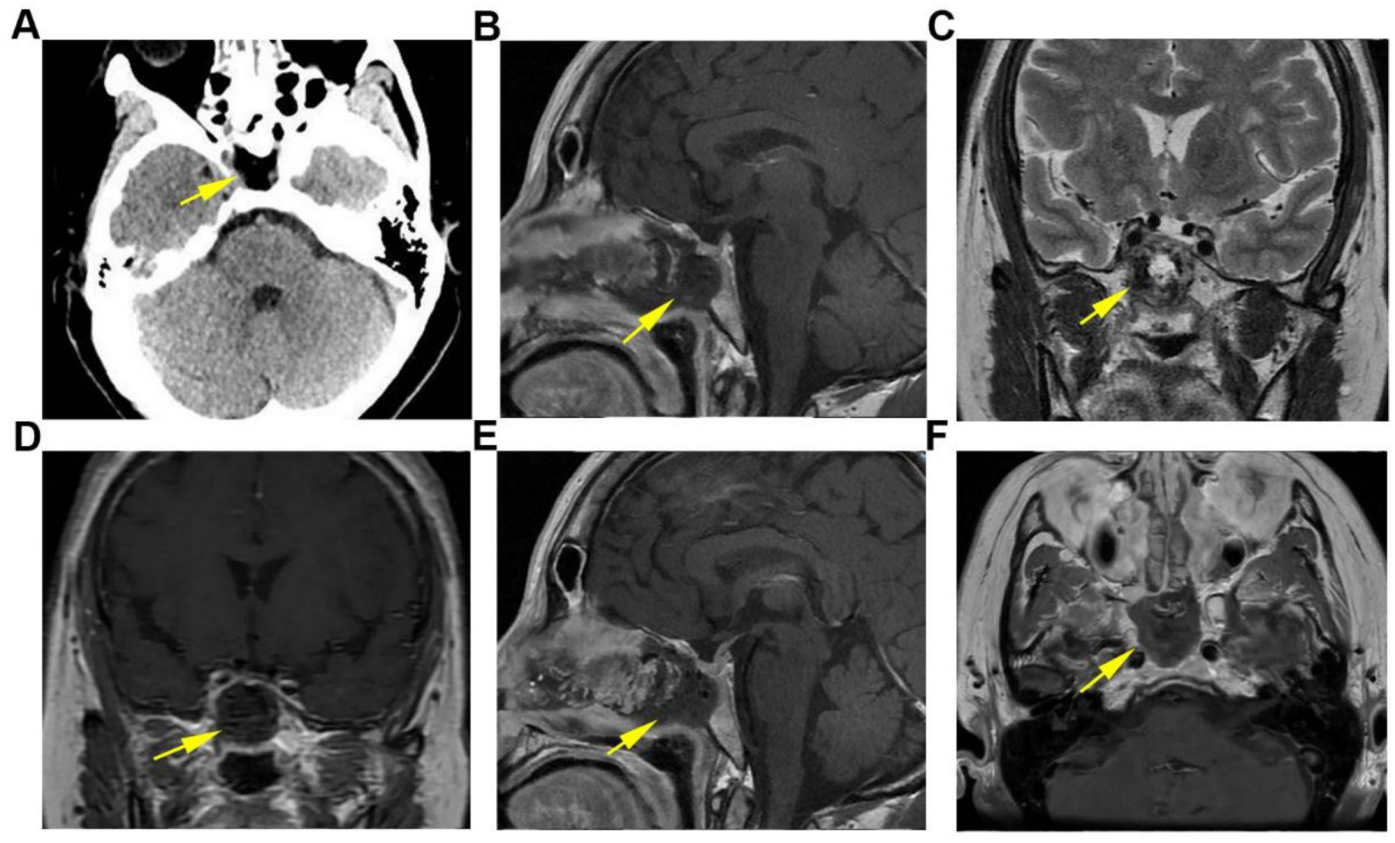
Postoperative imaging follow-up. **(A)** Axial non-contrast cranial CT image. **(B–E)** Cranial MR images: **(B)** Sagittal T1-weighted image. **(C)** Coronal T2-weighted image. **(D)** Coronal T1-weighted contrast-enhanced image. **(E)** Sagittal T1-weighted contrast-enhanced image. **(F)** Axial T1-weighted contrast-enhanced image. These images collectively indicate the complete resection of the lesion located in the sphenoid sinus region, with a well-healed surgical site and no discernible evidence of residual tumor.

**Figure 4:**
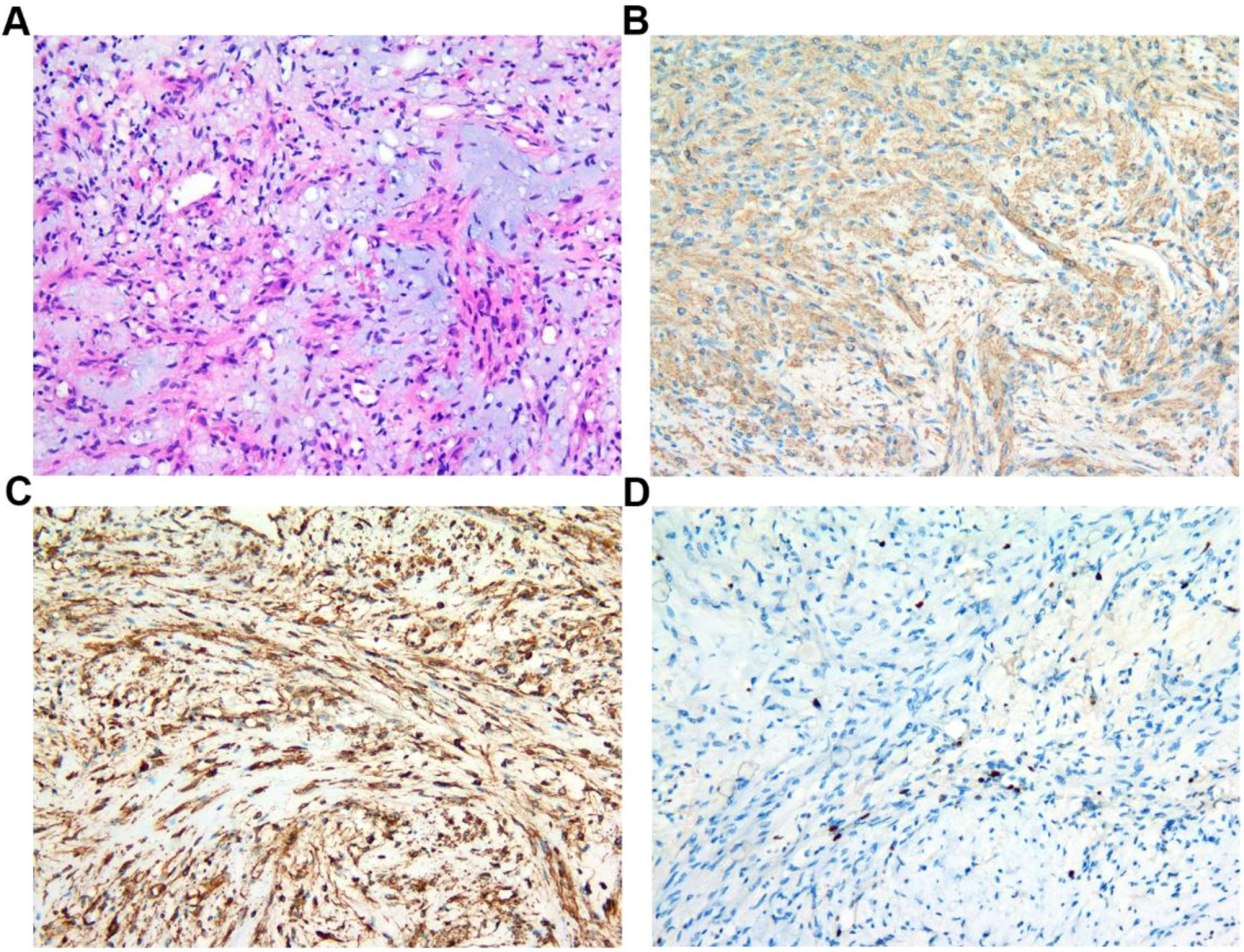
Postoperative pathology and immunohistochemistry. **(A)** Hematoxylin-eosin (H&E) staining reveals spindle-shaped neoplastic cells organized in fascicular patterns. **(B)** Smooth muscle actin (SMA) immunohistochemistry shows widespread strong positivity in tumor cells. **(C)** Vimentin immunohistochemistry reveals widespread strong positivity in tumor cells. **(D)** Ki-67 proliferation index is approximately 15%, indicating a relatively high proliferative activity of the tumor.

The patient’s postoperative recovery proceeded without complications, as there were no occurrences of cerebrospinal fluid rhinorrhea or intracranial infection. The preoperative conditions of ptosis and diplopia exhibited significant improvement. Subsequent cranial CT and MRI scans verified the complete resection of the tumor (**Figure 3**). The postoperative pathological diagnosis identified the tumor as a myofibroma. The immunohistochemical analysis yielded the following results: SMA(+), Vimentin(+), ERG (vascular +), CD34 (vascular +), S-100 (scattered positive cells), H3K27Me3(-), Desmin(-), Pan-CK(-), GFAP(-), EMA(-), PR(-), SSTR-2 (focal weak positivity), HMB45(-), MelanA(-), TFE3(-), STAT6(-), TRK(-), ALK(-), MUC4(-), Ki-67 (index 15%) (**Figure 4**). At the 3-month postoperative follow-up, the patient remained asymptomatic with complete resolution of ptosis and diplopia. Follow-up cranial MRI demonstrated no evidence of tumor recurrence, and the patient has returned to her normal daily activities. Continued long-term surveillance is planned to monitor for potential late recurrence.

## Discussion

Myofibroma is an infrequent benign neoplasm predominantly composed of myofibroblasts. While this tumor is more prevalent in pediatric populations, instances in adults have also been documented. In adults, myofibroma typically manifests as a solitary lesion, most frequently located in the skin and subcutaneous tissues of the head and neck, although it may also develop in other regions such as bone and muscle (7–9). The case of an adult myofibroma originating in the sphenoid sinus, as reported here, is clinically rare and presents with nonspecific symptoms. Clinical manifestations such as headache, visual impairment, and cranial nerve palsy can easily be misinterpreted as other sphenoid sinus pathologies, including invasive pituitary adenoma or sphenoid sinus mucocele (10–12).

In the context of imaging studies, both CT and MRI are instrumental in precisely delineating the tumor’s location, size, and its relationship with surrounding anatomical structures. CT scans are particularly effective in demonstrating bone invasion by the tumor; in this instance, the preoperative CT scan revealed destruction of the sellar floor bone. MRI, with its superior soft tissue resolution, provides detailed information on the tumor’s signal characteristics and its relationship with adjacent neurovascular structures (13). Contrast-enhanced MRI is essential for evaluating tumor vascularity. Myofibromas generally exhibit moderate to marked enhancement due to their rich vascularity. For instance, a myofibroma located in the jaw of a 7-year-old girl demonstrated marked enhancement on contrast-enhanced MRI (14).

In the present case, the sphenoid sinus myofibroma appeared as a round-like soft tissue density on CT, accompanied by partial erosion of the sellar floor. On MRI, the lesion demonstrated long T1 and long T2 signals, with marked enhancement following contrast administration. Imaging differential diagnosis should consider the following common skull base pathologies: ① Meningiomas: Typically present as iso-intense on T1-weighted images and iso- to slightly hyperintense on T2-weighted images, with homogeneous enhancement and the characteristic dural tail sign on MRI. On CT, meningiomas are often associated with hyperostosis rather than bone destruction (15–17); ② Invasive pituitary adenomas: Originate within the sella turcica with the epicenter located in the pituitary fossa rather than the sphenoid sinus, frequently encase the internal carotid arteries, and may show displacement of the normal pituitary gland (18); ③ Solitary fibrous tumors: Often exhibit a characteristic “flow-void” sign on T2-weighted images and demonstrate a progressive enhancement pattern on dynamic contrast-enhanced studies (19).

Histopathological examination remains the definitive method for diagnosing myofibroma. The diagnostic criteria encompass both the tumor’s histological morphology and its immunohistochemical profile. The histological morphology is distinguished by a biphasic pattern: pale regions comprising spindle-shaped myoid cells organized in nodules or short fascicles, and darker regions consisting of round or polygonal cells arranged in a hemangiopericytoma-like pattern (20). Immunohistochemical characteristics are crucial for diagnosis, with tumor cells typically expressing α-smooth muscle actin (α-SMA) and vimentin, while generally lacking expression of S-100 protein and CD34 (21). Accurate pathological diagnosis necessitates differentiation from other spindle cell tumors, such as solitary fibrous tumor, inflammatory myofibroblastic tumor, and schwannoma. Solitary fibrous tumors are typically characterized by the expression of CD34 and STAT6 (22, 23). Inflammatory myofibroblastic tumors frequently exhibit a significant inflammatory infiltrate, with a subset of cases demonstrating expression of the ALK protein (24). In contrast, schwannomas are characterized by the expression of the S-100 protein, a feature not commonly observed in myofibromas (25). The molecular biology underlying myofibromas is primarily associated with mutations in the PDGFRB gene and the aberrant activation of its related signaling pathways (26).

Surgical resection constitutes the primary therapeutic intervention for myofibroma. The fundamental principle guiding surgical management is the achievement of gross total resection under conditions that ensure patient safety. The surgical approach is contingent upon the tumor’s anatomical location and size. In cases where myofibroma is located within the sphenoid sinus cavity, the endoscopic endonasal approach is favored. Preoperative imaging is crucial and must be meticulously analyzed to evaluate the tumor’s relationship with adjacent anatomical structures, including any bone destruction and vascular encasement. During surgery, it is essential to achieve sufficient intratumoral decompression prior to the exploration of peripheral structures, with a focus on controlling hemorrhage and ensuring safe reconstruction of the skull base (27). The endoscopic endonasal transsphenoidal approach offers an optimal vantage point for addressing lesions situated in the central skull base. The successful outcome of this surgery hinged on several critical steps: First, leveraging the endoscopic wide-angle field and the advantage of proximity to clearly delineate the tumor’s relationship to the internal carotid artery, optic nerves, and cavernous sinus. Second, adopting a strategy of intratumoral piecemeal decompression to create the necessary space for safe marginal dissection. Third, thoroughly drilling away the skull base bone eroded by the tumor to pursue a radical resection. Fourth, executing a multilayer, watertight cranial base reconstruction using artificial dura mater, thereby effectively preventing postoperative CSF leakage.

Nevertheless, the infrequency of adult sphenoid sinus myofibroma has resulted in the absence of standardized treatment guidelines, thereby necessitating the development of individualized treatment strategies tailored to the specific clinical presentation of each patient. The necessity of adjuvant therapy following gross total resection remains a contentious issue, particularly in adult cases. Generally, the prognosis for myofibroma is favorable after complete surgical excision. However, this particular case, characterized by its occurrence in the sphenoid sinus—a deep anatomical location—and a high proliferation index (Ki-67 >5%), may present a relatively elevated risk of local recurrence. Radiation therapy in the management of sphenoid sinus myofibroma is primarily indicated for postoperative residual or recurrent tumors, as well as for cases where surgical resection is not feasible. Pharmacological therapy is less commonly employed, mainly reserved for cases where neither surgery nor radiotherapy is viable. Commonly used agents include non-steroidal anti-inflammatory drugs (NSAIDs), targeted agents, and chemotherapeutic drugs. Targeted agents are primarily directed at patients with PDGFRB mutations, with commonly used drugs including imatinib and sunitinib (28, 29). A case of pelvic myofibroma in a 35-year-old woman responsive to NSAID treatment has been reported (30).

The multidisciplinary team (MDT) for intrasphenoidal myofibroma includes neurosurgeons, pathologists, radiologists, radiation oncologists, and medical oncologists. The core function of the MDT is to formulate personalized treatment plans and enhance therapeutic outcomes (31). In this case of intrasphenoidal myofibroma, which involved a complex location, an MDT consultation was conducted after complete tumor resection. The pathologists confirmed the diagnosis of myofibroma. The medical oncologists recommended regular follow-up after discharge. Notably, the radiation oncologists suggested that adjuvant radiotherapy could be considered postoperatively, based precisely on the four high-risk features: “occurrence in an adult,” “deep-seated location,” “high Ki-67 index (15%),” and “bone infiltration.” Currently, although there is no high-level evidence supporting adjuvant radiotherapy following gross total resection, it may be utilized to further reduce the risk of local recurrence. However, given the generally favorable prognosis of completely resected myofibromas and the potential long-term risks of radiation to critical skull base structures, such decisions require extremely careful individualized consideration and thorough discussion with the patient. The advantage of MDT collaboration in this case lies in improving treatment efficacy and patient satisfaction.

The main challenges in prognostic assessment for intrasphenoidal myofibroma lie in its rarity and the lack of long-term follow-up data. Furthermore, prognostic evaluation indicators are not yet standardized. Future efforts should focus on establishing unified prognostic assessment criteria and conducting multicenter long-term follow-up studies to accurately evaluate the prognosis of intrasphenoidal myofibroma and clarify its optimal adjuvant treatment strategy. This study also has certain limitations. This report is a single-case retrospective review, with a relatively short follow-up period. The long-term efficacy and recurrence status require further observation.

## Conclusion

This study reports an extremely rare case of primary myofibroma originating within the sphenoid sinus cavity in an adult. The case clarifies the following key points: For myofibromas located in this complex central skull base region, the endoscopic endonasal transsphenoidal approach is a safe and effective minimally invasive surgical technique. It enables complete tumor resection, preservation of critical neurovascular structures, and reliable reconstruction of the skull base. The final diagnosis in this case relied on characteristic histopathological morphology and an immunohistochemical profile positive for SMA and Vimentin. Of particular importance, this case reveals that myofibromas occurring in deep locations, such as the sphenoid sinus, in adults may exhibit more aggressive clinicopathological features, such as bone destruction and a relatively high Ki-67 proliferation index (15%). This finding highlights the necessity for precise risk assessment based on individual pathological characteristics. Therefore, for such cases with high-risk factors, postoperatively establishing a multidisciplinary team comprising neurosurgery, pathology, radiation oncology, and medical oncology for comprehensive evaluation is of crucial guiding value. This process is essential for assessing recurrence risk, discussing the necessity of adjuvant therapies (such as radiotherapy), and formulating individualized long-term follow-up plans, constituting a key step in optimizing long-term patient outcomes. Future research should focus on multicenter collaboration and long-term follow-up to further clarify the natural history and optimal management strategies for adult sphenoid sinus myofibroma.

## Data Availability

The original contributions presented in the study are included in the article/Supplementary Material. Further inquiries can be directed to the corresponding author/s.
